# Higher Concentration of Marijuana Dispensaries in Neighborhoods with More Disadvantage Following Legalization in Washington

**DOI:** 10.21203/rs.3.rs-3393457/v1

**Published:** 2023-10-05

**Authors:** Edwina Williams, Pamela Trangenstein, Deidre Patterson, William Kerr

**Affiliations:** Alcohol Research Group; Alcohol Research Group; Alcohol Research Group; Alcohol Research Group

**Keywords:** Washington, Marijuana, Disadvantaged Neighborhoods

## Abstract

Washington is one of 21 states that have legalized recreational marijuana resulting in neighborhoods that have experienced a change in physical environment with the emergence of dispensaries. This study examines the selection of dispensaries into disadvantage area, incorporating local policies and neighborhood characteristics. Marijuana and alcohol sales data were from the Washington State Liquor and Cannabis Board; and neighborhood characteristics were drawn from the American Community Survey 2010–2016 5-year estimates. Using factor analysis we created a neighborhood disadvantage index where census tracts were stratified into disadvantaged tertiles; and counties were stratified by urban/rural status. We examined the association between dispensaries, neighborhood characteristics, and local marijuana policies using Negative Binomial Regression with a natural log of land area as an offset, separately for 2014–2016. Dispensaries opened in high-disadvantaged CTs in 2014 and then dispersed across the state while retaining higher concentrations in disadvantaged urban CTs. Marijuana-specific policies (allotted dispensaries and retail cap) were found to be predictors of marijuana dispensary density. This study provides evidence that marijuana dispensaries were disproportionately located in areas with greater disadvantage. State and local marijuana policies emerged as important predictors, underscoring the importance of designing thoughtful and equitable license allocation procedures and policies.

## Introduction

The Controlled Substances Act of 1970 made marijuana use and possession illegal in the United States under federal law. However, states have established their own policies and regulations concerning marijuana. Twenty-one states and Washington, DC have legalized recreational marijuana for persons 21 + years of age. Although marijuana remains federally illegal, use is common among adults and adolescents and more prevalent in Washington prior to legalization (Kerr et al., 2018; Subbaraman and Kerr, 2020). According to the 2021 National Survey on Drug Use and Health, approximately 13.0% of people aged 12 years or older (36.4 million people) used marijuana in the past month (Administration, 2022). Due to changes in policy and legalization, neighborhoods have experienced a change in physical environment, specifically the emergence of marijuana dispensaries. It is suggested that increased availability of marijuana, including dispensaries providing legal access, is associated with higher rates of marijuana use and a shift in social norms (Everson et al., 2019; Freisthler and Gruenewald, 2014; Martins et al., 2016).

In 2012, Washington State experienced two historic changes in substance use regulation within months of each other. First, Initiative-1183 (I-1183) ended the state monopoly of spirits sales on May 31, 2012, removing several state regulations related to distribution and pricing; and it established new spirits taxes including a 20.5% spirits volume tax paid directly by the consumer. Off-premise spirits outlets increased nearly five-fold from 333 outlets in 2012 to 1,600 in 2014. On average, spirits prices for 750mL containers increased 15% from 2012–2014 (Kerr et al., 2015) and 3.9% from 2014–2016 (Williams et al., 2020). The increased availability of alcohol and spirits was associated with increased violence in Seattle, WA (Tabb et al., 2016).

Second, Initiative-502 (I-502) legalized marijuana for recreational use effective December 6, 2012. I-502 authorized the Washington State Liquor and Cannabis Board (WSLCB) to govern and tax marijuana products for persons 21 and older, and to establish a new threshold for marijuana-impaired driving. Initially, retail marijuana license were limited to 334 applicants via a lottery. Following an analysis of the marketplace, the WSLCB increased the number of licenses to 556 in 2016. I-502 also authorized the WSLCB (state-level) and localities (county and cities) to develop marijuana-related policies, which may lead to an uneven distribution of dispensaries across the state. For example, the WSLCB established buffers for marijuana businesses restricting them from being within 1,000ft of places where children congregate, whereas some localities have reduced these buffers to 100ft.

Although recreational marijuana is becoming more available throughout the US, the impact of dispensaries on neighborhoods and communities remains unclear and much of what is known is based on studies of medical dispensaries. Prior studies have found evidence of an association between increased medical dispensaries and increased rates of use (Freisthler et al., 2015) and hospitalizations for marijuana abuse/dependence (Mair et al., 2015). Other studies have found dispensaries tended to be located in areas near alcohol outlets (Morrison et al., 2014; Thomas and Freisthler, 2016) which has severe implications on public health as these substances are often used together by the same people. The literature remains varied when examining the association between dispensaries and crime. In Colorado, availability of medical and recreational dispensaries was associated with higher rates of crime (Boggess et al., 2014; Shi et al., 2016). However, a study in Sacramento, California found no evidence of an association between medical dispensaries and crime (Kepple and Freisthler, 2012). Whereas a study in Long Beach, California found violent crime increased 1.5–4.8% for every increase of one medical dispensary per square mile (Freisthler et al., 2016).

To date only two studies have examined the spatial distribution of retail marijuana dispensaries in Washington. An analysis of Washington marijuana retail licenses in 2017 found positive associations with off-premise alcohol outlets and poverty but not with crime or ethnic diversity (Tabb et al., 2018). Another study that examined changes in marijuana retail density from 2014–2017 found retail density increased over time, and the most-deprived areas had an increased likelihood of retail density compared to the least-deprived areas (Amiri et al., 2019). The present analyses examines potential selection of marijuana dispensaries into disadvantages areas, incorporating local marijuana policies and neighborhood contextual characteristics. We hypothesize that areas without retail bans or stronger location restrictions, and those with higher disadvantage, disorder, urbanicity, minority density, alcohol outlets and crime will have more marijuana retail outlets.

## Materials and Methods

### Marijuana Dispensary and Alcohol Outlet Data

Data on marijuana dispensaries and off-premise alcohol outlets for 2014–2016 were obtained from the WSLCB. For dispensaries, we used the license number to link monthly sales and excise tax data to the list of marijuana applicants, which contained the license number, tradename, and address. There were 363 unique license numbers with reported sales activity over the study period, and 362 linked successfully to the license information. These dispensaries includes those with and without an endorsement to sell medical marijuana. Alcohol data contained the address for 5,618 outlets with an active license. The address for each dispensary and alcohol outlet was geocoded then spatially joined to the census tract (CT) polygon and summarized as the total count per CT per month using ArcGIS Pro (Esri, 2022).

### Sociodemographic and Crime Data

CT-level sociodemographic data for 2014–2016 were obtained from the American Community Survey (ACS) using Social Explorer. The following variables were derived from ACS: land area, total population, proportion of racial/ethnic minorities (percent Non-White), percent vacant housing units, percent occupied housing units, percent overcrowded (more than one person per room), percent population mobility (having moved within the past five years) and percentage of population stability (having lived in the same house for ten or more years). Using factor analysis, we created a neighborhood disadvantage index based on known key indicators (percentages): households with an income ≤$30,000, single-parent families, female-headed families, housing units without access to a car, people below 100% poverty, people receiving public assistance, and units renter occupied (Cronbach’s alpha: 0.8970). The composite score was then divided into tertiles (1 = low, 2 = medium, 3 = high). We classified urban/rural counties according to the Washington State Office of Financial Management’s definition – rural counties are those with a population density < 100 people per square mile or those < 225 square miles – for which, eight of 39 counties were urban.

Crime data were drawn from the ESRI crime risk index, a series of crime indices from the Applied Geographic Solutions calculated using FBI Uniform Crime Report databases. Crime risks includes personal crimes index (i.e. homicide, rape, robbery, and assault crimes) and property crimes index crimes (burglary, larceny, and motor vehicle theft crimes); these indices assess the relative risk for major crimes in each WA zip code. Further details on the crime risk methodology can be found elsewhere (ESRI Community Analyst, 2015). The values of each index were continuous ranging from zero to 577 for personal crimes and zero to 598 for property crimes. The crime data was linked to the CTs using a spatial join in ArcGIS Pro.

### Policy/Environment Data

We obtained monthly policy data at the city and county levels from Dilley et al. (2017). This database contains data on marijuana-related ordinances for Washington cities with ≥ 3,000 residents (142 cities) and all 39 counties, excluding policies related to federal lands. We incorporated the following variables into the analysis: number of retail licenses allotted by WSLCB (range: 0–42) and local retail cap on the number of retail license in the jurisdiction (yes/no)). City-level policies were primarily used in the analysis, when missing the county-level policy was utilized. Lastly, the presence of a major highway (yes/no) was determined by spatially joining the National Highway System for State Routes Shapefile to the CT layer in ArcGIS Pro.

### Analysis

This study examined the distribution of dispensaries in Washington at the CT level beginning in July 2014 – when retail sales first began – through December 2016. CT-months were the unit of analysis for this study because they are a proxy for neighborhoods. There were 1,445 residential CTs considered, but 45 CTs were considered islands (i.e. they did not share a border with another CT) and were removed to permit tests of spatial dependence resulting in 1,400 CTs.

First, we investigated the changing spatial distribution of dispensaries throughout the state using choropleth maps for each year. Next, we assessed the association between dispensaries, neighborhood characteristics, and local marijuana policies using Negative Binomial Regression models to account for over-dispersion. Models were run separately for each year in the study period (2014: n = 8,400; 2015 and 2016 n = 16,800) and stratified by urban/rural designations. We expected the number of dispensaries to be higher in CTs with more land area; therefore, we included the natural log of land area as an offset to correct for the unequal opportunities for dispensaries in CTs. All models were fit using STATA 17 with robust standard errors (StataCorp, 2021).

A row-standardized Queen Adjacency matrix defines neighboring CTs, where two CTs are considered neighbors if they share an edge. Moran’s Index (Moran’s I) calculated on the Pearson regression residuals tested for spatial dependence using R (Moran, 1950; R Core Team, 2021). Initially we ran Moran’s I on the residuals from a null model to determine whether the outcome contained spatial dependence in each month. We then repeated this process for the adjusted model to assess whether the regression coefficients explained the spatial dependence detected in the null analysis. Finally, we calculated an average Moran’s I value for each year of the analysis.

## Results

**Figure 1:**
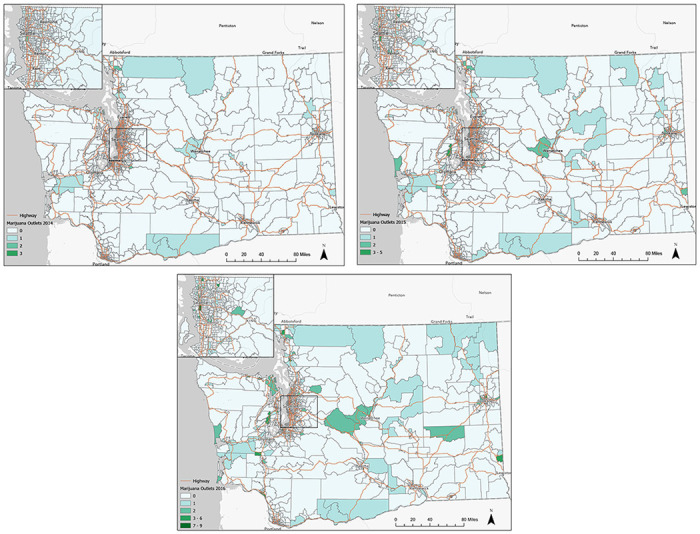
**a.** Choropleth maps of marijuana dispensaries in Washington state 2014. **b.** Choropleth maps of marijuana dispensaries in Washington state 2015. **c.** Choropleth maps of marijuana dispensaries in Washington state 2016.

Table 1 presents the descriptive statistics for the study variables averaged across the study period and stratified by urban/rural status. Figure 1 presents the choropleths maps of the total number of dispensaries per year, with King County enlarged for viewing purposes. In 2014, 86 dispensaries opened across 73 CTs concentrated in Seattle, Tacoma, Spokane, and Olympia. By 2016, the total number of dispensaries increased to 358 across 225 CTs. Moreover, most dispensaries opened in Western Washington, which is more urban than Eastern Washington. A single CT in Seattle contained the most dispensaries in 2015 (n = 5) and 2016 (n = 9).

### Regression Models

Results of the adjusted models for the association between dispensary density and sociodemographics, crime, neighborhood characteristics and marijuana policies are presented in Table 2. After averaging by year, no evidence of residual spatial dependence was found in the null or adjusted models, suggesting the CTs included in the models met the Negative Binomial assumption of independence. Significant associations were found between dispensary density and neighborhood characteristics across time, as described for urban and rural counties below.

### Urban Counties

There was a monotonic association between dispensary density and disadvantage across all three years in urban counties, although the strength of this association decreased over time. During the first year of the marijuana retail market in 2014, CTs with medium disadvantage had more than 50 times as many retailers (IRR = 56.55, 95% CI 17.95, 178.17; *P* < .001) compared to CTs with the lowest level of disadvantage. The association decreased to 7 times as many in 2015 (IRR = 7.43, 95% CI 5.48, 10.07; *P* < .001) and 4 times as many in 2016 (IRR = 4.30, 95% CI 3.32, 5.57; *P* < .001). When comparing the most disadvantaged tertile of CTs to the least advantaged CTs, greater neighborhood disadvantage was associated with having more than 120 times as many retailers in 2014 (IRR = 122.77, 95% CI 33.62, 448.33; *P* < .001). Similarly, the association decreased over time to 11 times as many retailers in 2015 (IRR = 11.25, 95% CI 7.63, 16.60; P < .001), and seven times as many retailers in 2016 (IRR = 7.36, 95% CI 5.28, 10.26; P < .001).

While percent of the population who was not White was insignificant in 2014, a 1% increase in the percent of non-White people was associated with a 2% increase in the number of marijuana outlets in 2015 and 2016 (2015: IRR = 1.02, 95% CI 1.01, 1.02, P < .001; 2016: IRR = 1.02, 95% CI 1.01, 1.03, P < .001).

The association between property crimes and dispensary density increased over the study period. In 2015 property crimes was associated with a 51% increase in retail density (IRR = 1.51, 95% CI 1.29, 1.76; *P* < .001) for every one-unit increase in the property crimes index and a 55% increase (IRR = 1.55, 95% CI 1.34, 1.80; *P* < .001) in 2016.

Dispensary density was greater among CTs that contained a highway compared to those that did not, although the magnitude declined over time. Beginning in 2015 CTs with a highway had 1.47 (95% CI 1.20, 1.80; *P* < .001) times as many dispensaries compared to CTs that did not have a highway; and by 2016 this association was reduced to 1.43 (95% CI 1.22, 1.68; *P* < .001) times as many dispensaries.

Off-premise alcohol outlets were also significantly associated with dispensary density across the study period. When retail dispensaries opened in 2014, a one-unit increase in off-premise alcohol outlets was associated with an 18% increase in retail density (IRR = 1.18, 95% CI 1.12, 1.24; *P* < .001). This association declined to an 11% increase in retail density (IRR = 1.11, 95% CI 1.09, 1.14; *P* < .001) in 2015, and a 10% increase in retail density (IRR = 1.10, 95% CI 1.09, 1.12; *P* < .001) in 2016.

Results revealed a positive association between the policy variables and dispensary density in the later years of the study period. For each additional dispensary allotted was associated with a 4–5% increase in retail density (2015: IRR = 1.05, 95% CI 1.01, 1.09; *P* = 0.01 and 2016: IRR = 1.04 95% CI 1.03, 1.04; *P* < .001). Lastly, now that the market has commercialized in 2016, CTs with a retail cap had approximately 1.77 (95% CI 1.43, 2.19; *P* < .001) times as many dispensaries, compared to CTs that did not have a retail cap.

### Rural Counties

A similar association between dispensary density and high area disadvantage observed in urban counties was also observed in rural counties. That is, across the study period CTs in the high disadvantage tertile had more dispensary retailers compared to CTs in the low disadvantage tertile, but the strength of this association decreased over time. When the market unfolded CTs in the high disadvantage tertile had 15 times as many dispensaries (95% CI 4.86, 49.33; *P* < .001), which declined to 3 times as many dispensaries (95% CI 2.01, 5.49; *P* < .001) by 2016. In 2014, CTs with medium disadvantage had approximately 5.86 (95% CI 2.05, 16.73; *P* < .001) times as many marijuana retailers, compared to CTs with less disadvantage. Over time, the direction of the association changed but the results were not significant.

Personal crimes and property crimes were found to have associations in the opposite directions for retail density in rural counties. At the opening of the retail market in 2014, a one-unit increase in the personal crimes index was associated with an 88% decrease in retail density (IRR = 0.12, 95% CI 0.03, 0.47; *P* < .001). In 2015, the association had declined to a 70% decrease in retail density (IRR = 0.30, 95% CI 0.15, 0.61; *P* < .001), but by 2016 the association rose to an 83% decrease in retail density (IRR = 0.17, 95% CI 0.10, 0.32; *P* < .001). Property crimes were found to be positively associated with retail density, where the magnitude of the association declined across the study period. In 2014, retail density increases by a factor of 16.57 (95% CI 6.88, 39.91; *P* < .001) for every one-unit increase in property crimes. By 2016, retail density was found to increase by a factor of 3.02 (95% CI 2.06, 4.44; *P* < .001) for every one-unit increase in property crimes.

In rural counties, evidence of a positive association between retail density and CTs that contained a major highway was found, where the magnitude of the association increased as the retail market commercialized. In 2014, CTs that contained a major highway compared to those that did not had approximately 2.39 (95% CI 1.08, 5.25; *P* = 0.03) times as many retail outlets. By 2016, the association had increased where CTs that contained a major highway had approximately 11.11 (95% CI 6.92, 17.84; *P* < .001) times as many retailers compared to CTs that did not contain a major highway.

The association between retail density and alcohol outlets was also found to increase across the study period. That is, in 2014 every one-unit increase in off-premise alcohol outlets was associated with a 7% increase in retail density (IRR = 1.07, 95% CI 1.01, 1.14; *P* < 0.01), among CTs in rural counties. The magnitude of this association continued to increase and by 2016 a one-unit increase in off-premise alcohol outlets was associated with a 30% increase in retail density (IRR = 1.30, 95% CI 1.25, 1.35; *P* < .001).

Lastly, the association between allotted number of dispensaries and retail density was found to increase across the study period in rural counties; however, the association was not significant in 2014. As the market commercialized, a one-unit increase in allotted number of dispensaries was associated with 5% increase in retail density (IRR = 1.05, 95% CI 1.01, 1.08; *P* < 0.01) in 2015, and an 11% increase in retail density (IRR = 1.11, 95% CI 1.08, 1.15; *P* < .001) in 2016. Although there was significant evidence of a negative association between CTs in areas with a retail cap compared to those without a retail cap, the magnitude of the association was minimal.

## Discussion

This study examined marijuana dispensary density in relation to neighborhood characteristics and local marijuana policies in Washington State from 2014 to 2016. Consistent with previous research (Amiri et al., 2019; Firth et al., 2020; Shi et al., 2016; Tabb et al., 2018), marijuana dispensaries were disproportionately located in CTs with greater disadvantage. Dispensaries appeared to open in CTs with particularly high levels of disadvantage in 2014 and then disperse across the state while retaining higher concentrations in disadvantaged urban CTs. The association between dispensary density and race depended on urbanicity. Marijuana dispensary density was higher in non-White CTs in urban areas but lower in such neighborhoods in rural settings. State and local marijuana policies emerged as important predictors, underscoring the importance of designing thoughtful and equitable license allocation procedures and policies.

A central finding was that marijuana dispensary density was higher in CTs with greater disadvantage, more people of color, and markers of disorder. This is consistent with previous analyses that concluded that recreational dispensary density was higher in CTs with more deprivation (Amiri et al., 2019), poverty (Tabb et al., 2018), people of color (Shi et al., 2016), and crime (Shi et al., 2016). However, our findings suggest that marijuana dispensaries are not indiscriminately opening in disadvantaged areas. Specifically, dispensary owners seem to avoid locations with violent crime and vacant properties that may deter customer business by making them feel unsafe. In addition, marijuana licensees may seek locations near off-premise spirits outlets, assuming the two businesses will share a customer base. Alternatively, the co-location of marijuana dispensaries and off-premise alcohol outlets may result from local governments using similar zoning and land use approaches to the two businesses (Thomas and Freisthler, 2016). Finally, the association with the presence of a highway suggests licensees prioritize locations with convenient access for customers. While our results describe dispensary locations, stakeholder interviews with licensees would complement these data to better understand selection of available land parcels. This information could inform efforts to equitably spread dispensaries across communities with consideration of business performance.

Several potential explanations for why dispensary density would be higher in disadvantaged CTs. The WSLCB allocation procedure that preferenced locations with more medical marijuana users may have inadvertently assigned more marijuana licenses to deprived CTs if marijuana demand was higher in them (Morrison et al., 2014). At a more local level, NIMBYism (“not in my backyard-ism”) may have also been at play, particularly in 2014 when the first dispensaries opened and associations with disadvantage were the strongest. NIMBYism often occurs when people organize to prevent adult entertainment (e.g., strip clubs, alcohol outlets) or otherwise stigmatized businesses (e.g., methadone treatment centers) from opening in their neighborhoods, often to allay fears about amenity harms and reducing amenity property values (Morrison, 2013). NIMBYism may occur by neighborhood residents protesting new marijuana licensing applications on a case-by-case basis, or it can be formalized via zoning and land use regulations or city/county bans/moratoria on marijuana dispensaries, which may disproportionately occur in locations with more resources (Matthay et al., 2022). As of 2016, 65% of WA residents lived in a city/county with marijuana zoning laws, and 30% lived in cities/counties with a permanent or temporary ban on marijuana sales (Dilley et al., 2017). Available commercial parcels that conform with local and state laws may be more common in deprived neighborhoods, as has been shown with alcohol outlets (Hippensteel et al., 2019). Given the racist history of zoning and land use policies, future research should explore whether common dispensary location policies, such as restricting them to commercial zones and establishing minimum distance requirements, could play a role in the disparate distribution of marijuana dispensaries. However, these policies are likely not the only explanation; an analysis that accounted for such land use and zoning provisions in Portland, Oregon concluded that there would be 73% more dispensaries in two otherwise equal neighborhoods that differed by one unit of disadvantage (Firth et al., 2020). Emerging evidence suggests that dispensary density is associated with marijuana use prevalence and frequency (Ambrose et al., 2021; Everson et al., 2019), including daily/near-daily use of adolescents and young adults (Shih et al., 2021). Considering that most dispensary sales are to daily/near-daily users (Kerr and Ye, 2022; Kilmer et al., 2014), the geographic patterning of marijuana dispensaries may hold public health consequences for those who live nearby.

After our study period, WA’s social equity program considered disadvantage directly when they awarded 44 licenses that were not issued, cancelled, or forfeited. A redress for the disparate burden of the war on drugs shouldered by communities of color, these equity-based licensing programs aim to allocate profits of the new legal cannabis sales to disproportionately impacted areas (Kilmer et al., 2021). WA defined social equity applicants as people who fulfilled at least two of the following three criteria: 1) lived in a disproportionately impacted area (e.g., high poverty, unemployment) for at least five years between 1980 and 2010, 2) was or had a family member arrested/convicted of a cannabis-related offense, and 3) had a household income less than the state median ($82,400). While access into this new industry ought to be equitable, it is unknown whether these programs will result in more dispensaries opening in disproportionately impacted areas, possibly unintentionally compounding existing harms.

As expected, policies shaped the distribution of marijuana dispensaries across WA. The WSCLB’s algorithm that originally allocated licenses to cities and counties granted more licenses to jurisdictions with higher rates of medical marijuana use (Caulkins and Dahlkemper, 2013). This suggests that states’ decisions about how to initially distribute licenses will determine what the marijuana retail environment looks like within their borders; these decisions ought to be considered carefully. City- or county-level thresholds on marijuana dispensary density were also associated with the number of dispensaries in the CT. These policies were associated with fewer marijuana dispensaries during the initial phase of legalization in both urban (2014) and rural (2014 and 2015) CTs. However, these limits were associated with more marijuana dispensaries in urban areas in 2016. While this may seem counterintuitive, this finding may suggest that these policies are in place in the urban areas with the greatest demand and most likely to encounter oversaturation in the future. It is also worth noting that four of the ten cities that passed thresholds on marijuana dispensary density set those thresholds at the number of licenses allocated by the WSLCB, while the other six were more restrictive (Dilley et al., 2017).

This study contains several limitations. First, while CTs are proxies for neighborhoods, they may not fully represent the neighborhood in which people live and dispensaries are located. Second, we only considered retail marijuana density. Future studies should also consider other dimensions of access, such as product and economic availability. Third, we utilized the OFM classification of rural counties. A more granular classification system with RUCA codes is useful for areas with similar characteristics, but the OFM county-level classification systems offered stability over time.

The marijuana industry is relatively new, yet rapidly growing. Currently, 21 states including Washington D.C. have legalized recreational marijuana use, yet the authors are aware of only four other studies on the distribution of recreational marijuana dispensaries in the U.S. to guide policymakers (Amiri et al., 2019; Firth et al., 2020; Shi et al., 2016; Tabb et al., 2018). There are a host of legislative, practical, and social equity concerns regarding recreational marijuana licensing that will determine how dispensaries distribute in communities and who bears the brunt of any harms that may manifest as a result. Marijuana licensing commissions ought to proceed cautiously and evaluate their efforts, such as by monitoring the number of licenses according to neighborhood disadvantage as the recreational market commercializes.

## Figures and Tables

**Table 1.** Descriptive Statistics of Study Variables Stratified by Urban Rural Status

	Urban				Rural				P-value
	Mean	SD	Min	Max	Mean	SD	Min	Max	
Dispensaries per Square Mile	0.10	0.53	0.00	11.31	0.04	0.24	0.00	5.09	<.001
Total Population	4927.21	1662.05	22.00	13302.00	473.77	2213.86	150.00	13122.00	<.001
Neighborhood Disadvantaged	1.94	0.82	1.00	3.00	2.22	0.75	1.00	3.00	<.001
% Non-White	28.81	16.86	0.00	94.73	27.15	22.14	0.00	96.44	<.001
% Home Owner	62.59	21.76	0.00	99.45	65.92	17.24	0.00	94.88	<.001
% Vacant Homes	6.66	4.58	0.00	54.10	14.28	12.76	0.00	87.20	<.001
% Overcrowded Homes	2.78	3.07	0.00	46.73	4.33	5.05	0.00	32.56	<.001
% Population Mobility	34.25	12.70	0.00	100.00	29.90	11.33	7.10	83.58	<.001
% Population Stability	53.54	19.29	0.00	100.00	58.01	18.97	0.00	91.01	<.001
Personal Crimes	0.82	0.74	0.00	5.77	0.72	0.34	0.00	1.71	<.001
Property Crimes	1.41	0.94	0.00	5.98	1.26	0.45	0.00	2.34	<.001
Contains Highway	0.60	0.49	0.00	1.00	0.74	0.44	0.00	1.00	<.001
Off-Premise Alcohol Outlets	3.56	3.72	0.00	31.00	5.10	4.30	0.00	21.00	<.001
Number of Retail Dispensaries Allowed	3.56	9.20	0.00	42.00	1.99	3.04	0.00	14.00	<.001
Retail Cap	0.03	0.17	0.00	1.00	0.03	0.17	0.00	1.00	0.98

**Table 2.** Incident Rate Ratios for Census Tract Characteristics and Retail Marijuana Dispensaries in Washington State 2014 - 2016

	2014			2015			2016		
	IRR	P-val	95% CI	IRR	P-val	95% CI	IRR	P-val	95% CI
**Urban**									
Total Population	**0.96**	**<.001**	**(0.95,0.98)**	**0.98**	**<.001**	**(0.98,0.99)**	**0.98**	**<.001**	**(0.98,0.99)**
Neighborhood Disadvantage (ref Low)									
Medium	**56.55**	**<.001**	**(17.95,178.17)**	**7.43**	**0.00**	**(5.48,10.07)**	**4.30**	**<.001**	**(3.32,5.57)**
High	**122.77**	**<.001**	**(33.62,448.33)**	**11.25**	**<.001**	**(7.63,16.60)**	**7.36**	**<.001**	**(5.28,10.26)**
% Non-White	0.99	0.26	(0.97,1.01)	**1.02**	**<.001**	**(1.01,1.02)**	**1.02**	**<.001**	**(1.01,1.03)**
% Home Owner	1.02	0.11	(1.00,1.04)	1.00	0.60	(0.99,1.01)	**0.99**	**0.02**	**(0.98,1.00)**
% Vacant Homes	**0.88**	**<.001**	**(0.83,0.93)**	**0.88**	**<.001**	**(0.86,0.90)**	**0.90**	**<.001**	**(0.88,0.91)**
% Overcrowded Homes	0.93	0.20	(0.84,1.04)	**0.86**	**<.001**	**(0.83,0.89)**	**0.88**	**<.001**	**(0.85,0.90)**
% Population Mobility	**1.04**	**0.05**	**(1.00,1.08)**	**1.09**	**<.001**	**(1.03,1.14)**	**0.98**	**0.02**	**(0.96,1.00)**
% Population Stability	1.02	0.22	(0.99,1.06)	1.05	0.06	(1.00,1.11)	**0.97**	**<.001**	**(0.95,0.98)**
Personal Crimes	1.13	0.61	(0.70,1.82)	0.88	0.26	(0.70,1.10)	**0.62**	**<.001**	**(0.50,0.78)**
Property Crimes	1.27	0.23	(0.86,1.86)	**1.51**	**<.001**	**(1.29,1.76)**	**1.55**	**<.001**	**(1.34,1.80)**
Contains Highway (yes/no)	1.52	0.13	(0.89,2.61)	**1.47**	**<.001**	**(1.20,1.80)**	**1.43**	**<.001**	**(1.22,1.68)**
Off-premise Alcohol Outlets	**1.18**	**<.001**	**(1.12,1.24)**	**1.11**	**<.001**	**(1.09,1.14)**	**1.10**	**<.001**	**(1.09,1.12)**
Number of Retail Dispensaries Allowed	0.74	0.09	(0.51,1.05)	**1.05**	**0.01**	**(1.01,1.09)**	**1.04**	**<.001**	**(1.03,1.04)**
Retail Cap (yes/no)	**<0.01**	**<.001**	**(<0.01, <0.01)**	0.28	0.23	(0.04,2.21)	**1.77**	**<.001**	**(1.43,2.19)**
Moran’s I	0.00	0.34		0.02	0.12		0.02	0.18	
**Rural**									
Total Population	0.99	0.20	(0.98,1.00)	**0.99**	**0.04**	**(0.98,1.00)**	**0.98**	**0.00**	**(0.98,0.99)**
Neighborhood Disadvantage (ref Low)									
Medium	**5.86**	**<.001**	**(2.05,16.73)**	0.76	0.19	(0.51,1.14)	0.85	0.40	(0.59,1.24)
High	**15.48**	**<.001**	**(4.86,49.33)**	**7.28**	**<.001**	**(3.94,13.44)**	**3.32**	**<.001**	**(2.01,5.49)**
% Non-White	**0.95**	**<.001**	**(0.93,0.97)**	**0.95**	**<.001**	**(0.93,0.96)**	**0.97**	**<.001**	**(0.96,0.99)**
% Home Owner	1.01	0.61	(0.98,1.04)	**1.04**	**<.001**	**(1.01,1.06)**	1.01	0.15	(0.99,1.03)
% Vacant Homes	**0.89**	**<.001**	**(0.86,0.92)**	**0.94**	**<.001**	**(0.92,0.95)**	**0.93**	**<.001**	**(0.92,0.94)**
% Overcrowded Homes	1.03	0.43	(0.95,1.12)	**1.04**	0.16	**(0.98,1.10)**	0.99	0.77	(0.93,1.05)
% Population Mobility	1.21	0.24	(0.99,1.06)	0.83	0.23	(0.83,1.05)	**0.87**	**<.001**	**(0.84,0.91)**
% Population Stability	**0.94**	**0.01**	**(0.90,0.98)**	**0.78**	**<.001**	**(0.78,0.95)**	**0.83**	**<.001**	**(0.80,0.86)**
Personal Crimes	**0.12**	**<.001**	**(0.03,0.47)**	**0.30**	**<.001**	**(0.15,0.61)**	**0.17**	**<.001**	**(0.10,0.32)**
Property Crimes	**16.57**	**<.001**	**(6.88,39.91)**	**4.55**	**<.001**	**(2.85,7.26)**	**3.02**	**<.001**	**(2.06,4.44)**
Contains Highway (yes/no)	**2.39**	**0.03**	**(1.08,5.25)**	**7.21**	**<.001**	**(4.54,11.43)**	**11.11**	**<.001**	**(6.92,17.84)**
Off-premise Alcohol Outlets	**1.07**	**0.01**	**(1.01,1.14)**	**1.20**	**<.001**	**(1.16,1.25)**	**1.30**	**<.001**	**(1.25,1.35)**
Number of Retail Dispensaries Allowed	1.03	0.62	(0.91,1.16)	**1.05**	**0.01**	**(1.01,1.08)**	**1.11**	**<.001**	**(1.08,1.15)**
Retail Cap (yes/no)	**0.00**	**<.001**	**(0.00,0.00)**	**0.08**	**<.001**	**(0.04,0.18)**	0.58	0.15	(0.28,1.21)
Moran’s I	0.03	0.15		0.04	0.16		0.03	0.20	
